# Silver Micro-Nanoparticle-Based Nanoarchitectures: Synthesis Routes, Biomedical Applications, and Mechanisms of Action

**DOI:** 10.3390/polym13172870

**Published:** 2021-08-26

**Authors:** Md Abdul Wahab, Li Luming, Md Abdul Matin, Mohammad Rezaul Karim, Mohammad Omer Aijaz, Hamad Fahad Alharbi, Ahmed Abdala, Rezwanul Haque

**Affiliations:** 1 Institute for Advanced Study, Chengdu University, Chengdu 610106, China; liluming@cdu.edu.cn; 2 School of Science, Technology and Engineering, University of the Sunshine Coast, Sippy Downs, QLD 4556, Australia; rhaque@usc.edu.au; 3 Department of Pharmacy, NUB School of Health Sciences, Northern University Bangladesh, Globe Center, 24 Mirpur Road, Dhaka 1205, Bangladesh; mamatin76@yahoo.com; 4 Center of Excellence for Research in Engineering Materials (CEREM), Deanship of Scientific Research (DSR), King Saud University, Riyadh 11421, Saudi Arabia; mkarim@ksu.edu.sa (M.R.K.); maijaz@ksu.edu.sa (M.O.A.); harbihf@ksu.edu.sa (H.F.A.); 5 K.A. CARE Energy Research and Innovation Center, Riyadh 11451, Saudi Arabia; 6 Mechanical Engineering Department, College of Engineering, King Saud University, Riyadh 11421, Saudi Arabia; 7 Chemical Engineering Program, Texas A&M University at Qatar, Doha POB 23874, Qatar; ahmed.abdalla@qatar.tamu.edu

**Keywords:** antibacterial, pathogens, infectious diseases, silver nanoparticles

## Abstract

Silver has become a potent agent that can be effectively applied in nanostructured nanomaterials with various shapes and sizes against antibacterial applications. Silver nanoparticle (Ag NP) based-antimicrobial agents play a major role in different applications, including biomedical applications, as surface treatment and coatings, in chemical and food industries, and for agricultural productivity. Due to advancements in nanoscience and nanotechnology, different methods have been used to prepare Ag NPs with sizes and shapes reducing toxicity for antibacterial applications. Studies have shown that Ag NPs are largely dependent on basic structural parameters, such as size, shape, and chemical composition, which play a significant role in preparing the appropriate formulation for the desired applications. Therefore, this review focuses on the important parameters that affect the surface interaction/state of Ag NPs and their influence on antimicrobial activities, which are essential for designing future applications. The mode of action of Ag NPs as antibacterial agents will also be discussed.

## 1. Introduction

With the emergence of various infectious pathogens that show resistance toward one or more antibiotics, the treatment of patients becomes very difficult, eventually leading to deaths [1,2,3,4,5,6]. These various microorganisms include bacteria, protozoans, infectious agents, fungi, viruses, and prions. More critical is that foodborne, waterborne, and airborne pathogens can also enter the body via different modes of infection [4,6,7]. Over a million deaths per annum worldwide are reported due to these harmful infectious pathogens [6]. Pathogens such as *Escherichia coli* (*E. coli*) and *Staphylococcus aureus* (*S. aureus*) and viruses, including norovirus and influenza virus, are the most reported. Such infectious pathogens can vary in virulence, contagiousness, mode of transmission, and degree of severity of the infection [5,6,7]. To get rid of the infections associated with pathogens, antibiotics are successfully used to treat infected patients. Still, unfortunately, the excess use of antibiotics has increased antibiotic resistance and, broadly, antimicrobial resistance [8].

Meanwhile, combined treatments, such as the use and overuse of antibiotics and other associated antimicrobial agents, have shown a higher level of multi-drug antibiotic microbial resistance (known as MDR), resulting in low treatment efficacy [9]. Therefore, the rise of resistance to harmful pathogenic microorganisms has received huge attention globally from researchers, pharmaceutical manufacturers, and medical professionals [10]. For example, a person already shown MDR will be treated with a spectrum of antibiotics even if recovery is slow. Therefore, the additional antimicrobial resistance characteristics of different pathogens will bring a severe threat to the world [11,12,13]. Based on the pandemic and epidemic diseases report made by the World Health Organization (WHO), the death rate for the infection with drug-resistant pathogens was high [12]. Under these circumstances, health science requires new types of disinfection systems [1,2,3].

In this context, with the development of nanoscience and nanotechnology, particularly, Ag NPs have become one of the most prominent disinfection systems as a disinfectant in biomedical science for several decades [1,2,3,4,5]. Therefore, recently, the design and development of Ag NP-based antimicrobial agents with better efficacy have received huge attention [1,2,3]. Ag NPs with unique characteristics including excellent control of size and shape, loading, and antibacterial activities have emerged. Some studies have even shown that the size and shape can control antibacterial activities better [14,15,16,17,18,19]. Therefore, the modifications of Ag NPs from bulky to micro-size and then to nano-size scale along with designed shapes, sizes, and targeted compositions have been found to extend the bactericidal activities of Ag NPs [20,21,22,23,24,25,26,27,28,29,30,31,32,33,34,35]. Furthermore, the surface chemistry of Ag NPs has a crucial role in their potency because of the influence exerted from physical and chemical phenomena [36,37,38,39,40,41,42,43,44,45,46].

**Figure 1:**
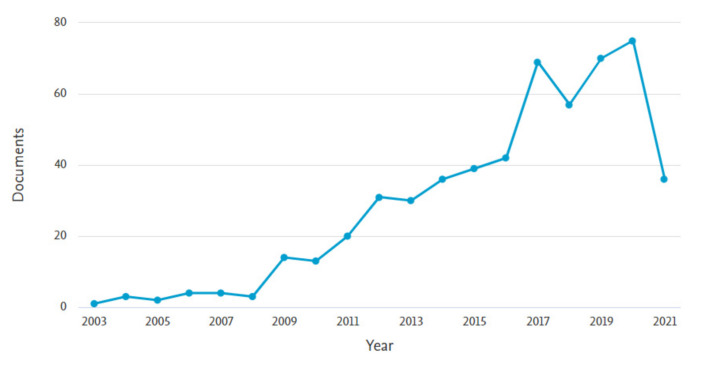
The number of annual publications on the topic of interest showing exponential growth (created using Scopus database [47]).

A Scopus survey [47] with the keyword ‘*silver nanoparticle*’ revealed 55,309 documents. The authors added a keyword, ‘synthesis’, with the Logic operator ‘AND’ to narrow this down. After this, 18,182 documents were revealed. To narrow down the literature further, the authors added another keyword, ‘*biocompatible*’, with the ‘*AND*’ logic operator. This ultimately revealed 549 documents from the year range of 2003–2021. The analysis of the search results is shown in Figure 1 and Table 1. More and more researchers are involved in this topic, and the research interest is increasing every day. As a result, an exponential trend is observed in the published document (Figure 1).

It is also evident from Table 1 that a significant number of review articles have been published on this topic (44 out of 549). However, only a handful of reviews were on silver nanoparticles [48,49,50,51,52]. Although various aspects were reviewed, a review article focused on the formation with different shapes and sizes, and the on-demand release of silver nanoparticles is highly demanded.

This review focuses on the synthesis of Ag NPs by considering some individual potential factors: synthesis routes and their impact on the particle size, shape, aggregation, and composition and their antimicrobial activities will also be discussed. The mode of action and side effects of Ag NPs will be demonstrated in this review.

## 2. Synthesis of Ag NPs

Several synthesis methods were reported for preparing nanoengineered Ag NP-based antimicrobial agents. Significantly, the types of particles will directly affect the final properties, which are highly associated with applications [53,54,55,56,57,58,59]. For example, Ag NPs capped with galactose and mannose have lower toxicity against hepatocytes and neuronal-like cell lines than NPs stabilized with citrate [60]. It is expected that nanoparticles (NPs) with better biocompatibility are more suitable for biomedical fields [61,62,63,64,65]. Therefore, the judicious choice of the Ag NP synthesis method is crucial to produce Ag NPs with special features for desired applications, particularly biomedical applications. However, the following existing methods will be briefly discussed in the following sections as they are predominantly used to prepare Ag NPs.

### 2.1. Wet Chemical Synthesis Route

The conventional wet chemical routes allow the preparation of Ag NPs with different sizes and shapes through chemical transformations of the Ag precursor by varying the experimental conditions. This route consists mainly of a few materials such as a Ag metal source, solvent, stabilizing/capping agent, and reducing agent [66,67]. The reduction is usually carried out with some reducing agents such as hydrazine, citrate of sodium, and sodium dodecyl sulfate. The reduction of Ag NPs also consists of two steps: nucleation and the growth of particles.

**Scheme 1:**
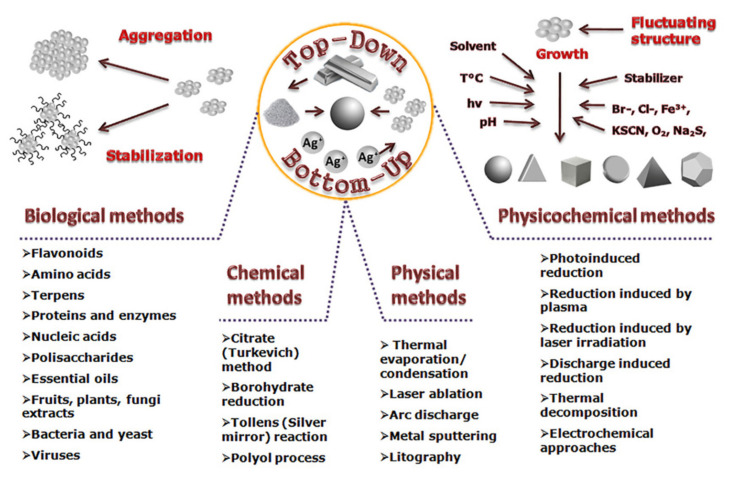
The synthesis scheme of Ag NPs via various methods GO (adapted with permission from reference [67]).

The chemicals used to synthesize Ag NPs such as citrate, borohydride, thioglycerol, 2-mercaptoethanol, solvents, and substrates are toxic and hazardous [68,69,70]. Additionally, the reaction will form byproducts. Some of them are toxic and harmful to the living system. Based on the reported literature, the preparation methods of Ag NPs could be broadly classified into (i) “Top-down” and (ii) “Bottom-up” methods [68,69,70] as shown in Scheme 1. “Top-down” processes use mechanical force to grind the bulk of the Ag source; then, the particle stabilization is carried out via some colloidal protecting agents [68,69,70]. The “Bottom-up” processes include chemical reduction, electrochemical routes, and sonolytic decomposition, also known as the wet chemical process. The advantage of these chemical routes is the high yield of Ag NPs [71,72,73,74,75]. These routes produce particles on which surfaces are sedimented with chemicals, which require further steps to remove them. On the other hand, it is hard to prepare Ag NPs with a well-controlled size and nonaggregated morphology. Therefore, an additional step is needed to prevent the aggregation of particles [76,77,78,79,80].

### 2.2. Physical Synthesis Routes

The physical synthesis routes mainly use force/pressure (mechanical grinding), temperature, electromagnetic radiation, and energy [67,81,82,83,84,85,86,87,88]. The advantages of physical routes are speed, minimal chemical consumption, and no hazardous agents involved. Still, they require high energy for the process and produce an extensive range of particles with low yield [67,68]. The “Top-down” approach is usually based on physical synthesis routes, which are less expensive, fast, and simple techniques than wet chemical routes. For example, the arc discharge is often employed for pure Ag atomization for forming Ag NPs [81]. Based on various studies, the shape and size of Ag NPs can easily be controlled by employing different synthetic conditions and media [84]. The synthesis medium has a significant impact on the shape, size, distribution, and aggregation of Ag NPs. Additionally, oxygen might help form the unexpected Ag_2_O instead of metallic Ag NPs, even by using an Ar jet at the interaction zone. Other methods, including sputtering, plasma during gas-phase synthesis, and lithography, are also used to prepare Ag NPs. Among these methods, lithography offers better control over the shape and size [82]. It should also be noted that lithography is a highly labor-intensive process that will provide rather expensive products.

### 2.3. Green/Biological Synthesis Route

Recently, the bioinspired method for the preparation of Ag NPs has become an alternative process to other reported methods because it is cost-effective, facile and straightforward, reliable, and more environmentally friendly. Therefore, tremendous attention has been devoted to preparing Ag NPs with various sizes and shapes using different biological approaches [89,90,91,92,93,94,95]. For example, Ganaie et al. [80] have reported the green synthesis of Ag NPs with controlled shape and size using weed mimosa. The process is more economical and could improve the economics of the production of Ag NPs. In the meantime, various kinds of Ag NPs were reported via green and biocompatible approaches without chemicals, including toxic agents [58,88,96,97,98,99,100,101,102]. These studies have suggested alternative preparing methods for the synthesis of Ag NPs, which largely depend on the following factors: the solvent, the reducing agent, and the non-toxic material. The great benefit of these biological approaches is the availability of various functional groups, including amino acids, proteins, or secondary metabolites, and a vast array of biological resources.

In contrast, the disadvantage of these methods is the additional steps needed to prevent the formation of nanoparticle aggregation. Overall, the preparation of Ag NPs was found to be very eco-friendly, cost-effective, and pollution-free in comparison to chemical-dependent processes. These methods produce NPs with a controlled size and shape [88,97,98,99,100,101,102], which are critical factors that should be considered for different biomedical-based applications.

### 2.4. Size and Shape That Contribute to the Inhibition of Bacterial Growth

Many studies have shown that the type of Ag NPs, including in terms of their size, shape, and concentration, has a significant impact on the inhibition process of the growth of bacteria [1,2,3,67,68,69,88,97,98,99,100,101,102]. For example, those Ag NPs having a similar active surface area will display how the shape of the particle can inhibit the growth of bacteria. Additionally, the active facets of the employed nanoparticles show different interactions with microorganisms. The wet chemical method mediated two Ag NPs that were prepared: (i) spherical of 15–90 nm and (ii) triangular of a ~150 nm size. Spherical particles of 15–90 nm have better antibacterial performance than triangular particles of a ~150 nm size against *Pseudomonas aeruginosa* and *Escherichia coli* [55]. Therefore, results have suggested that size does matter to penetrate bacterial cells [53,55,57] effectively. The spherical Ag NPs dominated by the (111) facets have effectively killed bacteria compared to the triangular-shaped Ag NPs [55,102], suggesting that each facet interacts differently with the bacterial surface [53,57]. Additionally, the highly reactive facets (111) with Ag NPs of <10 nm in size can effectively kill the bacteria cells via tagging Ag NPs into the sulfur-containing membrane [53], which is consistent with the previously reported antibacterial studies that focused on three sizes of stable Ag NPs (5, 15, and 55 nm mean values) tested against various bacteria [16,57,59].

### 2.5. Role of Support and Stabilizer

Ag NPs with different shapes and sizes have already displayed strong antibacterial activity against infectious pathogens, but particle instability in different media remains challenging. The size and aggregation of Ag NPs show dependency on the type of employed media. The stability of NPs is enhanced by using suitable stabilizing agents, which can prevent the agglomeration and precipitation of NPs during synthesis [60,103,104,105,106,107,108]. Among the stabilizers, polyvinyl pyrrolidone (PVP) is more effectively employed for controlling the size and agglomeration of NPs [109,110,111,112,113,114]. Therefore, its presence as the stabilizing agent can enhance their final functional activities because of structural advantages. The homogeneous dispersion of Ag NPs can enhance the antibacterial activity of pure supports such as carbon nitride against infectious pathogens [115]. Roman et al. [116] have reported Ag NPs under different polymeric stabilizers. The study suggested that 0.01% of sodium carboxymethylcellulose (CMC) can form better nanoparticles. Another study was conducted by Jung et al. [117] on the effects of polymeric stabilizers for the formation of various Ag NPs. Based on the coordination of Ag^+^ ions with PVP and poly(4-styrene sulfonic acid-co-maleic acid) sodium salt (PSSMA), it is suggested that PSSMA has better controllability over the final morphology of nanoparticles [117].

### 2.6. The Functional Properties of Ag NPs

Physical and chemical properties of Ag NPs are highly dependent on size and shape, distribution, type of morphology/facet, surface chemistry, surface area, composition, aggregation, and Ag^0^/Ag^+^, and all these factors will play a role in antibacterial activities. Additionally, the type of employed reducing agent and synthesis methods for preparing Ag NPS will have a significant role in determining cytotoxicity and inhibiting bacterial growth [61,63,118,119,120,121,122,123,124,125,126,127,128,129,130,131]. Various kinds of Ag NPs can easily be manipulated by different synthesis routes that will control the shape and sizes and impact the final materials’ toxicity, particularly for biomedical applications [132,133,134,135,136,137,138]. Studies have shown that particles with smaller dimensions can produce more toxicity than those larger particles employed because they have a larger surface area and due to their ability to penetrate the cell/membrane of bacteria [1,2,3,17,18,19,26,132,133,134,135,138]. The role of types of shapes and facets cannot be ruled out for controlling the toxicity of Ag NPs [26,133,134,135]. Suresh et al. [134] reported the comparative analysis on the effects of different surface coatings to prepare various Ag NPs via chemical and or biological coating methods. Results suggested that either method on the NPs surface can significantly affect the toxicity [134]. Ag NP surface charges also determine the toxicity effect in cells. More prominently, Tabata et al. [135] have reported that positive charges of NPs can stay for a long time in the bloodstream compared to negatively charged NPs [66,135], which is a major route for the administration of anticancer agents reported by other groups of authors [26,135].

## 3. Applications

Ag NPs have already become known as broad-spectrum antimicrobial agents, and their antibacterial activities depend on the nature and type of Ag NPs. However, Ag has been used by humankind for many years [1,2,3]. For example, Ag plays a significant role against some infections developed by infectious pathogens.

### 3.1. Burn and Wound Healing

**Figure 2:**
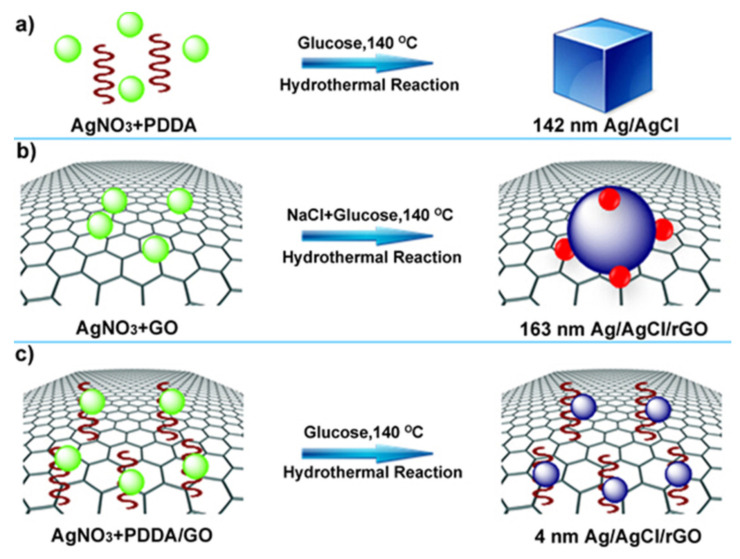
Schematic illustration of the size and shape of Ag/AgCl NPs controlled by (**a**) the PDDA surfactant, (**b**) GO sheets, and (**c**) both PDDA and GO (adapted with permission from reference [63]).

**Figure 3:**
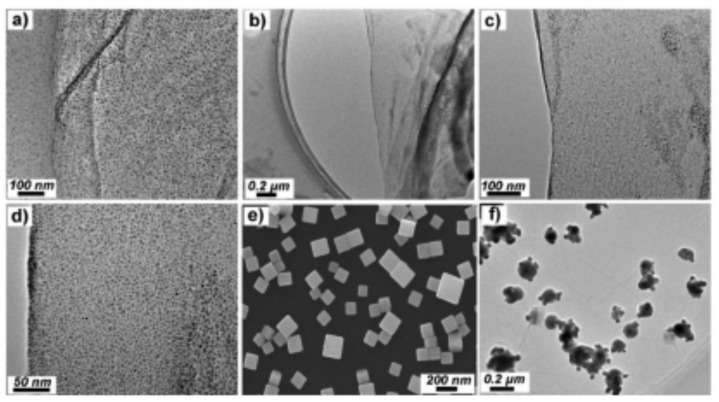
TEM images of Ag NPs: (**a**) Ag/AgCl/GO and (**b**–**d**) Ag/AgCl/rGO synthesized in the presence of GO and PDDA, (**e**) scanning electron microscope (SEM) images of Ag/AgCl synthesized in the presence of the PDDA surfactant, and (**f**) TEM image of Ag/AgCl/rGO synthesized without PDDA (adapted with permission from reference [63]).

**Figure 4:**
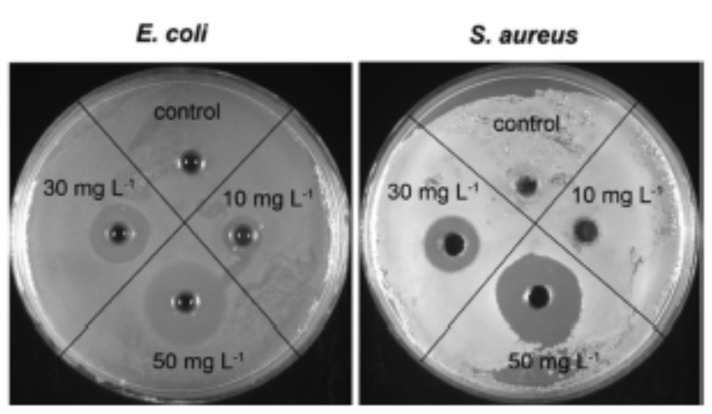
Photographs of *E. coli* and *S. aureus* grown around a series of concentrations of Ag/AgCl/rGO nanomaterials on the plates (adapted with permission from reference [63]).

**Figure 5:**
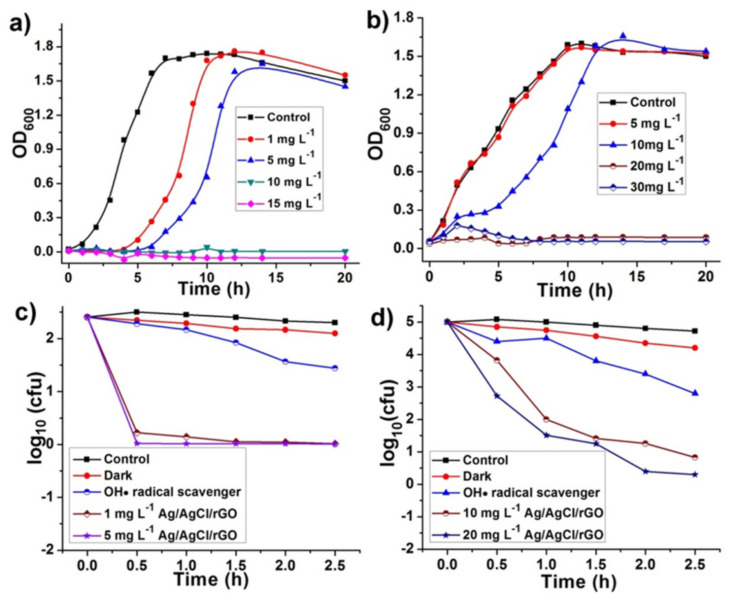
Growth profile in LB medium with serial concentrations of Ag/AgCl/rGO nanomaterial added to the culture of (**a**) *E. coli* and (**b**) *S. aureus*. Bacteria time–kill profiles within 2.5 h for (**c**) *E. coli* in the presence of 1 or 5 mg L^−1^ Ag/AgCl/rGO nanomaterial and (**d**) *S. aureus* in the presence of 10 or 20 mg L^−1^ Ag/AgCl/rGO nanomaterial. The bacteria time−kill profiles of *E. coli* (**c**) and *S. aureus* (**d**) treated with Ag/AgCl/rGO were also performed in a dark environment or with the OH* radical scavenger (adapted with permission from reference [63]).

The applications of Ag NPs are not limited to these: medical, antiseptic sprays, wound dressings, and thin antibacterial coatings on the surface of medical devices for preventing infection against infectious pathogens [1,2,3,8,9,56]. For example, Zhou et al. [63] have reported graphene-supported Ag/AgCl nanoparticles under various conditions in Figure 2 and Figure 3 against antimicrobial activity, and burn wound healing was assessed. Figure 2 also shows how to control the size and shape of Ag/AgCl NPs using the poly(diallyl dimethylammonium chloride) (PDDA) surfactant and graphene oxide (GO) sheets. Both PDDA and GO are also included in Figure 2. In this study, the presence of oxygen in GO functions as a site for coating the Ag NPs and prohibited the formation of the agglomeration of Ag NPs. The attached PDDA surfactant molecules on GO further enhanced the prevention of the aggregation of Ag NPs, which was confirmed by an image in Figure 3f. Therefore, the synergistic ability from both PDDA and GO has enabled the ultrafine-sized Ag/AgCl NPs in Figure 3. Among the images in Figure 3, Figure 3a shows that particles with less than 10 nm are uniformly distributed over the surface of GO sheets. In contrast, Figure 3b,c confirm the homogenous distribution of 4 nm Ag NPs on the rGO sheets facilitated by ultrasonication, which is well-consistent with previous reports on how the employed method impacts the formation of metal nanoparticles on the support [115]. These NPs were tested for antibacterial performance and suggested for the burn wound healing process, as displayed in Figure 4 and Figure 5 against Gram-negative and Gram-positive pathogens.

The antibacterial performance of the Ag/AgCl/rGO against *Escherichia Coli* (*E. coli*) and *Staphylococcus aureus* (*S. aureus*) was evaluated qualitatively in Figure 4. The Ag/AgCl/rGO-based agent has shown antibacterial activity against both bacteria based on the presence of inhibition zones. The growth kinetics profile of the employed pathogens in the Luria−Bertani (LB) medium in Figure 5 has confirmed the dose-dependent antibacterial effect of the Ag/AgCl/rGO. The minimum inhibitory concentrations (MICs) for Ag/AgCl/rGO in Figure 5 against *E. coli* and *S. aureus* were found to be 2 and 4 mg L^−1^, respectively, and the complete inhibition was reported at 10 mg L^−1^ for *E. coli* and *S. aureus* 10 mg L^−1^. The result from the biocompatibility is at <5 mg L^−1^, while antibacterial activity against both bacteria performed well. On the other hand, the burn wounds of healthy mice were treated with the Ag/AgCl/rGO sample for two weeks. A negligible scab was noted for Ag/AgCl/rGO-treated mice, while the control showed a big scab, indicating that the Ag/AgCl/rGO-treated burn wound had a fast-healing rate and epidermis regeneration.

**Figure 6:**
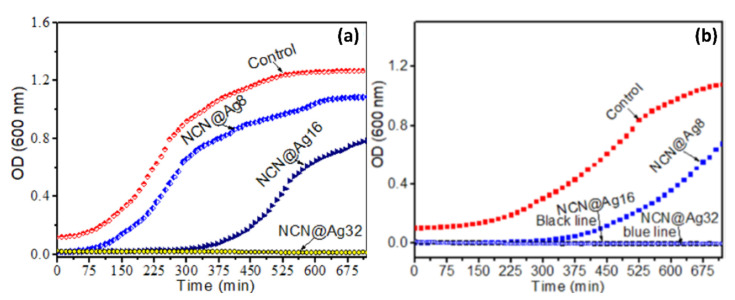
(**a**) Growth profiles of wild-type *E. coli* and (**b**) of MDR *E. coli* strains in the presence of control and various concentration of NCN@Ag NPs (adapted with permission from reference [115]).

Recently, Wahab et al. [115] used an in situ ultrasonication process to incorporate Ag NPs along with a single C-N precursor into the pore size (PS) (9.17 nm) of SBA15 silica to prepare highly nanoporous carbon nitride (NCN)-supported Ag NPs (NCN-Ag NPs). The resulting Ag NPs with less than 8 nm in NCN were tested against both wild type and multidrug-resistant *E. coli* pathogens, as shown in Figure 6.

Pure NCN was less effective against both pathogens, but NCN-supported Ag NPs have shown excellent antibacterial performance against both pathogens tested. It was reported that the complete inhibition at 32 μg/mL for the wild type and 16 μg/mL for the multidrug-resistant strain was achieved, indicating that MDR *E. coli* was found to be more susceptible in the presence of NCN@Ag NPs than wild-type *E. coli*. It should be noted that NCN alone could not show such antibacterial performance, but the presence of homogeneously dispersed Ag NPs in the NCN support efficiently inhibited the growth of both bacteria.

Microbial infection becomes a serious complication when it occurs in burn and wound sites [139,140,141,142,143]. In this context, Ag NPs/Ag NP-based composites play an essential role in wound healing/dressing applications [139,140,141,142,143]. Pat et al. [140] have reported Ag NPs (5–12 nm)/bacterial cellulose (BC) composites (Ag/BC) by the photochemical reduction process using UV radiation against *E. coli* for wound-healing [140]. The minimum amount of Ag NPs incorporated in BC showed the maximum ability to kill bacteria, even for a longer amount of time. The specific amount of Ag released after a specific time indicated the stability of the Ag NPs within BC and reduced the risk of toxicity if applied in wound healing [140]. Recently, Jin et al. [141] investigated the possible applications of the synergistic and on-demand release of Ag NPs-AMPs (antimicrobial peptide) incorporated into porous silicon for antibacterial and wound healing processes. In this study, the wound site was loaded with a Ag NPs-AMP@PSiMPs composite. The results have shown outstanding in vivo bacteria-killing activity, accelerated wound-healing, and low biotoxicity in an *S. aureus*-infected rat wound model [141]. Based on the results, the authors have suggested that this research could be useful as an on-demand release to fight wound infection and promote wound healing. More studies on Ag-NPs-based composites were reported [139,140,141,142,143].

### 3.2. Eco-Friendly and Biocompatible Application

Recently, green synthesis-mediated Ag NPs have been more preferred for biomedical applications than physical and chemical route-mediated Ag NPs because NPs from green synthesis are more eco-friendly, safe, biocompatible, and effective. For example, Azizi et al. [61] synthesized Ag NPs using hydrogel beads based on k-Carrageenan. Briefly, the Ag NPs were synthesized in aqueous *Citrullus colocynthis* seed extract. *Citrullus colocynthis* was used as both a reducing and capping agent, and cross-linked k-Carrageenan/Ag NPs hydrogel beads were also fabricated with the assistance of KCl. Green synthesis-mediated Ag NPs were effective as an antibacterial agent against *S. aureus*, Methicilin Resistant *S. aureus*, *P. aeruginosa*, and *E. coli*, with maximum zones of inhibition of 11 ± 2 mm.

**Figure 7:**
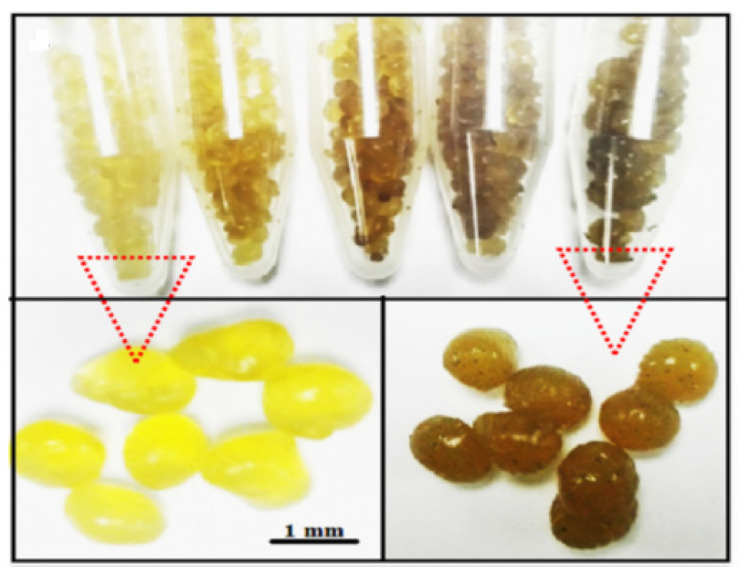
Digital image of Kappa-Carrageenan/Ag bio-nano composite with the various ratios of Ag-NPs (adapted with permission from reference [61]).

**Figure 8:**
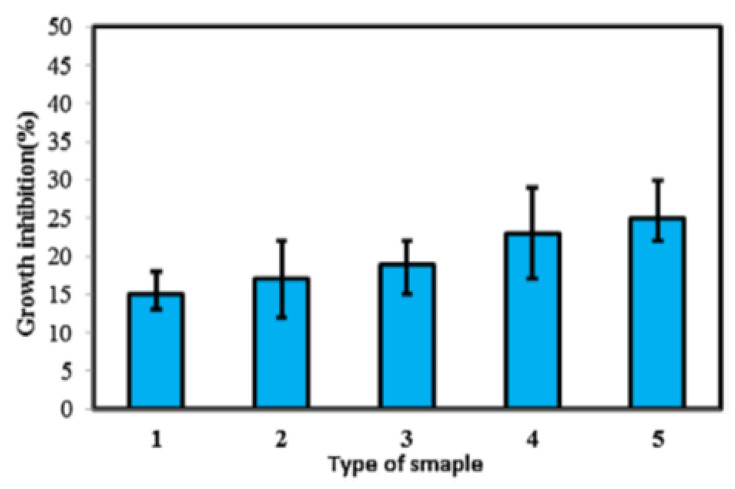
Cytotoxic effect of hydrogel Kappa-Carrageenan/Ag nanocomposites on the growth inhibition of VERO cells (adapted with permission from reference [61]).

Figure 7 shows how Ag NP concentration affected color, which changed from dark yellow to dark brown [61]. FESEM studies confirmed the formation of spherical Ag NPs of less than 25 nm. This study also conducted cytotoxicity as shown in Figure 8. The values of growth of inhibition at 1000 μg/mL were found to be much lower than 50%, indicating that the employed bio-nano composites do not have any toxicity effect, damaging elements for living cells. This could be a new avenue where hydrogels with the reported level of toxicity should impact pharmacological potential.

Among the studied pathogens, the antibacterial activity of PP-Ag NPs has shown the highest inhibition against *E. coli* (16.33 ± 0.14 mm) at a 40 μL concentration. In addition to the antibacterial application, free radical scavenging, anticancer, larvicidal, anti-acetylcholinesterase, reusability, and durability have also been assessed, and an associated discussion is available in [61]. 

### 3.3. Enhancing Tribological Properties

Recently, alloy nanoparticles of metals have become popular in biomaterial applications since combining two metals can provide enhanced functional activities, including stability, chemical, and tribological properties [65,120]. Vijayalakshmi et al. [119] have used various stabilizing agents to form a Ag@SnO_2_ core–shell. The antibacterial activity of chalcone dendrimer stabilized Ag@SnO_2_ core–shell NPs was tested against multiple pathogens, including *Bacillus subtilis*, *Proteus mirabilis*, *Candida albicans*, and *Aspergillus niger*. Interestingly, antibacterial activity against the Bacillus subtilis was more effective among the tested pathogens, whereas it did not show any activity against *Aspergillus niger*. In contrast, it acted mildly as an antibacterial agent against *Proteus mirabilis* and *Candida albicans*.

**Figure 9:**
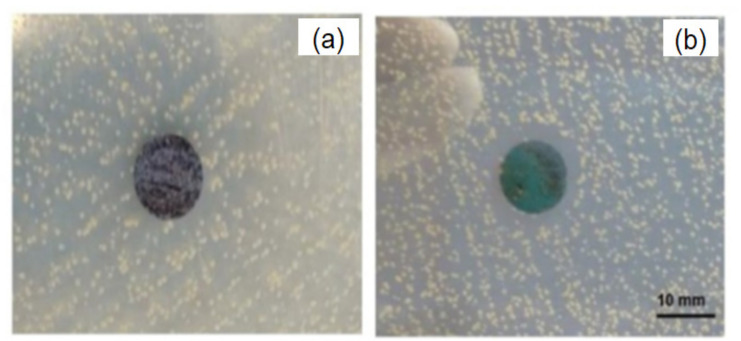
Inhibition experiments: images for 355–500 um after silanization and before Ag NP coating (**a**) and after Ag NP coating (**b**). Ten millimeter scale bar for both images (adapted with permission from reference [120]).

Torres et al. [120] demonstrated Ag NP-coated Ti porous substrates and dense particles against *S. aureus*, as shown in Figure 9, using a substrate of 40% of porosity [120]. Based on the inhibition experiment, particles with porosity are more effective toward antibacterial activity because more Ag NPs can be embedded into the wall of large pores of the porous Ti substrate.

**Figure 10:**
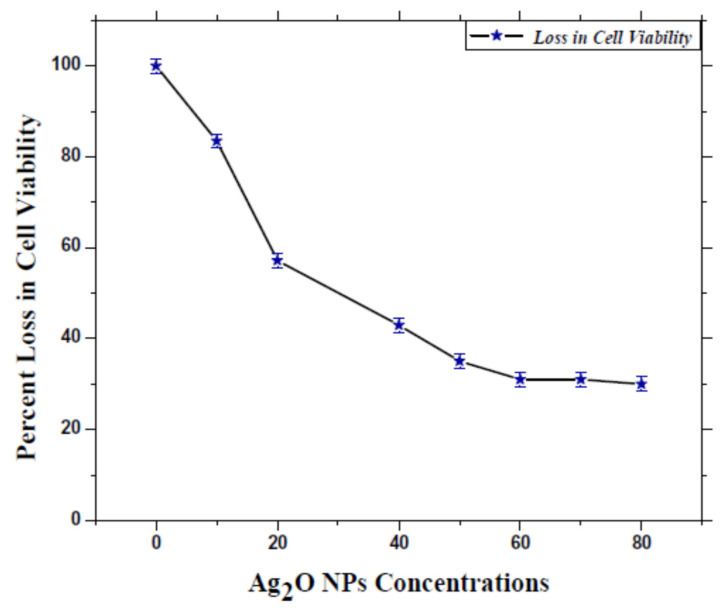
Percent cell viability loss in HepG2 cell viability (adapted with permission from reference [64]).

Iqbal et al. [64] demonstrated Ag_2_O NPs with a dominated crystallite size of 64.3 nm from the plane (111) via the wet chemical route. To support their use as an anticancer agent, cellular adsorption, phototoxicity analysis, and reactive oxygen species (ROS) analysis were systematically carried out. Figure 10 shows how the concentration of Ag_2_O NPs has impacted the percentage loss in cell viability.

**Figure 11:**
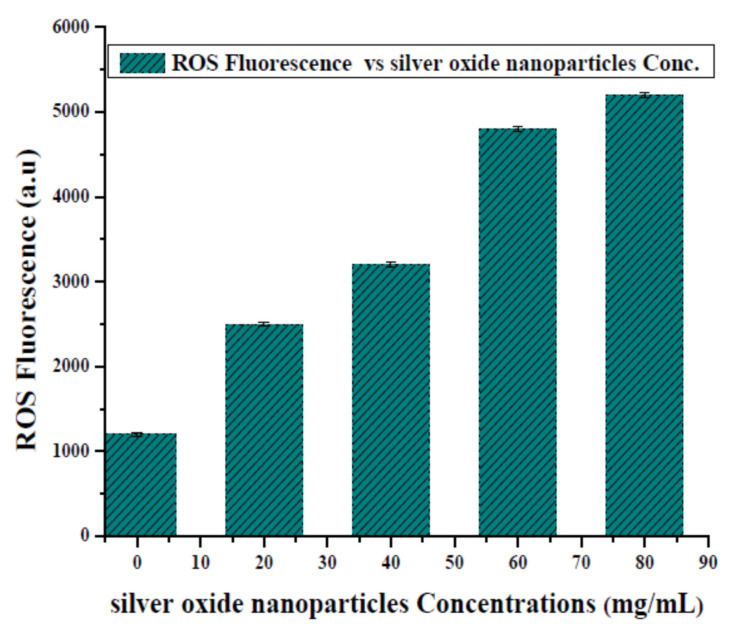
ROS fluorescence (a.u) for silver oxide nanoparticles exposed in in vitro HepG2 model (adapted with permission from reference [64]).

The significant loss in cell viability occurred when the concentration of Ag_2_O NPs was 60 mg/mL. Afterward, no change in the loss in cell viability is found, indicating that the optimal concentration of Ag_2_O NPs is (60 mg/mL) [64]. Figure 11 displays the ROS study results using the fluorescence which was found to depend on the employed concentration of Ag_2_O NPs [64]. The obtained results suggested that after a certain concentration, Ag_2_O NPs are found to be very toxic, which might be helpful for biomedical and clinical applications, as indicated by the authors. Based on the various analysis, Ag_2_O NPs have bio-interaction characteristics and physicochemical properties as an anticancer agent. This study was limited to spherical particles of 64.3 nm, but any analysis that can be carried out on less than 64.3 nm along with different particle facets might have more interesting results.

### 3.4. Aerobic and Anaerobic Environmental Activities

**Figure 12:**
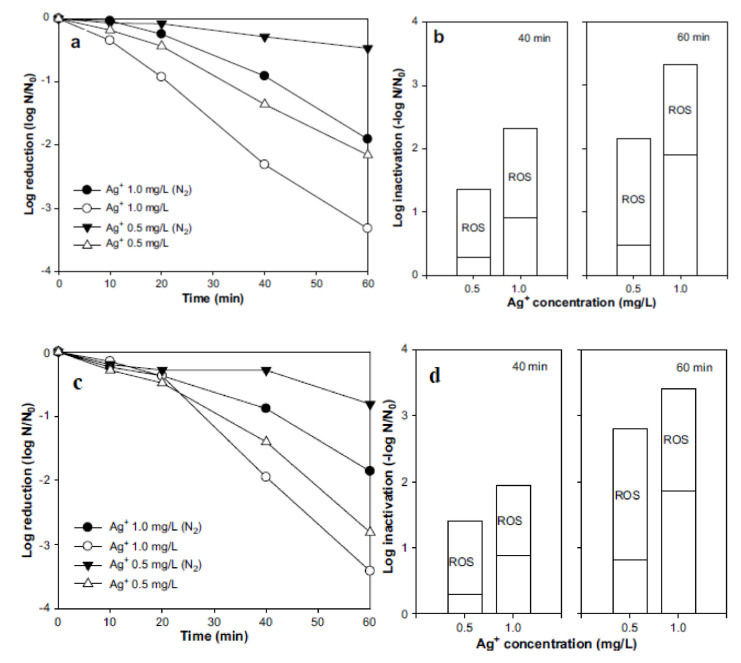
(**a**) The effect of Ag ions on the inactivation of *E. coli* ATCC8739 in the absence or presence of oxygen. (**c**) The effect of silver ions on the inactivation of *S. aureus* ATCC6538. (**b**,**d**) The proportion of ROS-mediated inactivation by silver ions at 40 and 60 min (*N*_0_ = 10^6^ CFU/mL, pH 7.1) (adapted with permission from reference [118]).

Interestingly, Park et al. [118] have reported ROS generation by Ag ions in the presence or absence of oxygen. Their anti-bactericidal activity was well-compared against *E. coli* and *S. aureus* in Figure 12. For the aerobic environment, 0.5 mg/L Ag ions required 2.2 log inactivation of *E. coli* in 60 min. In contrast, the same dose of Ag ions affected ~0.5 log inactivation for the anaerobic environment. As seen in Figure 12b, aerobic conditions have inactivated more efficiently than anaerobic conditions, suggesting that in the presence of O_2_, Ag ions killed bacteria effectively due to the production of superoxide radicals. This study has suggested that superoxide-radical-independent antibacterial activity is due to the thiol-interaction mechanism of Ag ions. Under the same conditions, a similar antibacterial activity was demonstrated against *S. aureus* in Figure 12c,d and suggested that Ag ions’ superoxide-radical-facilitated extra inactivation ability towards killing bacteria could be a general phenomenon [118].

**Figure 13:**
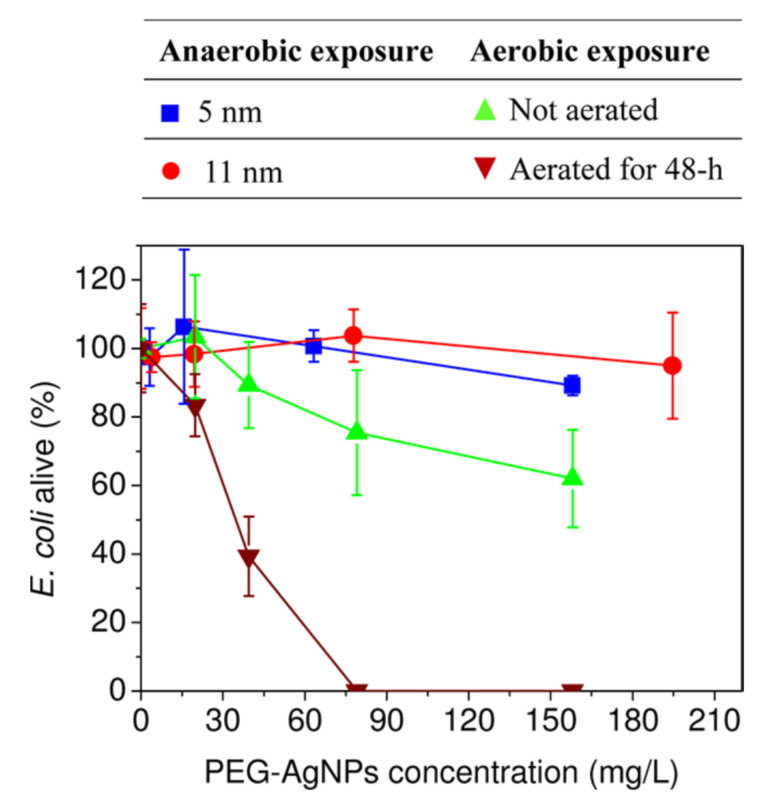
Antibacterial action of Ag NPs under aerobic and anaerobic atmosphere (adapted with permission from reference [121]).

Xiu et al. [121] systematically demonstrated the antibacterial activity of Ag NPs through manipulation of oxygen availability, particle size, shape, and/or type of coating. As depicted in Figure 13, it is evident that the presence of oxygen indeed has a significant impact on the killing of *E. coli*, which is consistent with other reported works [118].

According to the toxicity assay, aerobic conditions enhanced the toxicity of the system. Therefore, Ag^+^ ions released during the toxicity assay showed a significant antimicrobial effect, and even prolonged air exposure showed more impact on antibacterial toxicity of Ag NPs of 5 nm. These results evidenced that the toxicity of Ag NPs is quite sensitive to oxygen availability, and oxidative dissolution of the crystalline cores can result under an aerobic environment and increase the concentration of soluble Ag^+^ ions, which are needed to have a significant impact on the antibacterial activity [118,121].

### 3.5. Important Parameters That Control the Mechanism of Action

Based on the available literature [1,2,3,4,5,122,123,125,126,127,128], since the 1800s, Ag NPs have been used for food handling, currency, and preventing wound infections. Recently, the applications of Ag NPs have been tremendously expanded into various emerging advanced biomedical areas. Most of the reported studies have described the antimicrobial activity of Ag NPs due to the presence of Ag in the system. The addition of Ag NPs into the growth media of pathogens/bacteria will push Ag NPs towards the membrane of bacteria and possibly form the aggregation of Ag NPs that will slowly deteriorate the integrity of the bacterial membrane. Finally, cell death will occur [8,58,59].

**Figure 14:**
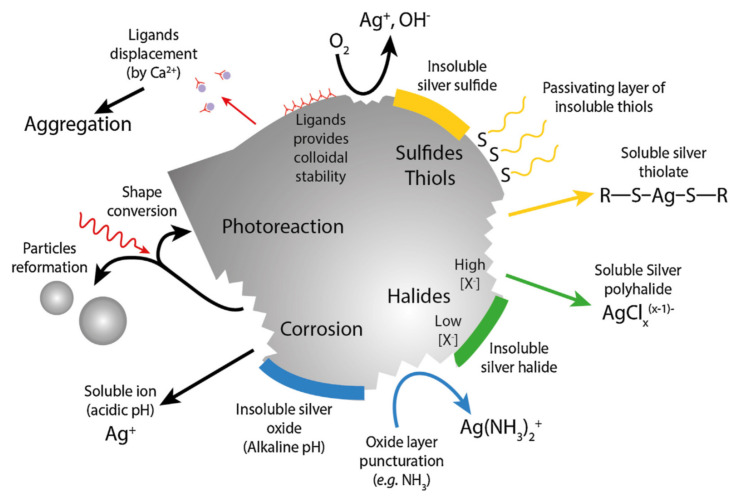
Summary of the factors affecting Ag NP dissolution and inhibition of growth of bacteria (adapted with permission from reference [5]).

Additionally, the overall mechanism of the antibacterial activity largely depends on the interaction between pathogens employed by Ag NPs. The particle size controls the interaction since small particles have a different interaction with the bacterial membrane than larger particles [8,58,59,121]. Figure 14 shows several phenomena affecting Ag NP dissolution, which will inhibit the growth of bacteria [5].

Since the first few reports of Ag particles, most of the studies have used the following factors that helped to develop the mode of action against antibacterial activity, which are highly related to each other [1,2,3,8,9,56]. Based on the above discussion, we can summarize the following factors already discussed for the mechanism of action.

(i) One of the main components is the design of the effective and potent antibacterial system that influences the overall mode of action since the mode of action of Ag NPs against pathogens is highly dependent on some factors such as modification, aggregation, size, shape, dissolution, ROS production, the release of adsorbed silver species, adsorption or desorption of ions, and the nature of polymers/supports/surfaces of supports [1,2,3,8,9,56].

(ii) The type of silver species (Ag^0^/Ag^+^) can be produced when a Ag source is added into the growth media of bacteria because the production of Ag particles in the media will first penetrate the cell wall of bacteria and react with peptidoglycans [125,127]. It is also noted here that the degree of interaction between the cell wall of bacteria and Ag NPs will be primarily controlled by previously mentioned parameters such as size, shape, aggregation, and type of Ag species already existing in the media.

(iii) The binding of Ag NPs to the cell wall of bacteria will release Ag^+^ ions that will show toxicity to kill the bacteria. Ag NPs can easily bind to membrane proteins, which can significantly affect the membrane’s permeability and lead to the breakage of cells [130]. Additionally, Ag NPs that bind to membrane proteins can significantly affect the uptake and release of phosphate ions, thus disrupting the respiratory chain and energy production [130].

(iv) Smaller particles can bind more effectively to the membrane and penetrate the cell wall of bacteria, and final inhibition of transcription occurs. The penetration of Ag NPs into the cell is also associated with intracellular structural components such as lipids, proteins, and DNA [130]. More clearly, they can damage DNA and act on protein synthesis [129].

(v) The produced ROS will affect cell membrane disruption and DNA modification. As the process goes on, the continuous release of Ag NPs’ Ag+ ions is more and more considered to be the mechanism of destroying infectious pathogens [128]. Thus, the result of cell death is a consequence of their action from this process [129].

### 3.6. Side Effects of Ag NPs

Since the 1800s, the applications of Ag NPs have been expanded in different areas, including environmental, biomedical, and domestic appliances. Moreover, recently, the development of nanomedicines using NPs is one of the promising strategies to combat various diseases, including infectious pathogen-caused diseases. Therefore, research on Ag NPs is carried out with different shapes and sizes, and how they impact users should be investigated thoroughly since each type of Ag NP particle shows not only a different level of toxicity but also antibacterial activity, which is highly related to size, shape, and concentration [1,2,3,10,67]. It is found that a smaller size of particles has shown better penetration ability. For example, if higher doses release more Ag NPs in aqueous media, this might lead to severe health complications [1,2,3,8,9,56]. Thus, it could be fairly suggested that caution in their use and further investigation of the mechanism of action based on available employed Ag NPs are necessary for avoiding long-term consequences. Based on the literature, it could be fairly suggested that Ag NPs could reach the body’s vital organs (such as lungs, intestine, liver, and so on) through the skin as nanosilver may penetrate more easily than micro/macro-silver particles. Eventually, the availability of such small nanoparticles might sometimes create some toxicity around the organs, leading to serious complications [128,144,145,146].

## 4. Conclusions and Outlook

Bacterial infections have become one of the main health concerns because more than a million deaths worldwide per annum are the result of these highly infectious pathogens. It is found that the absence of effective treatments will lead to various types of serious health complications. In addition, MDR is becoming another key problem in clinical scenarios. In this context, the use of Ag NPs at the nanoscale could provide an opportunity for antibacterial treatments because the nanosized effects and exotic behavior of Ag NPs have produced some outstanding characteristics including biological properties related to biomedical applications.

The research reviewed in this article has comprehensively demonstrated synthesis routes and their impacts on the formation of particles, shapes, and biomedical applications of Ag NPs, particularly focusing on the antibacterial activity of various infectious pathogens and their mechanisms. As discussed, because of multi-drug resistance, both researchers from academia and industry have explored Ag NPs as a possible alternative broad-spectrum antibacterial agent for reducing the risk of microbial infections. Hence, the design and development of Ag NP antibacterial agents over the last several years has been found to be one of the promising approaches to combatting diseases caused by various microorganisms. It is noteworthy to mention that Ag NPs of less than 10 nm are found to be more effective in inhibiting the growth of bacteria.

If this becomes true, it will help nanoscience and nanotechnology researchers to develop safer, biocompatible, and efficient antibacterials based on Ag NPs. Nonetheless, studies dealing with Ag NPs’ biocompatibility of interaction with cells and tissues are inevitable to avoid risks to human health as well as the environment since the overdose of Ag NP-based agents could have long-term adverse effects on both humans and the environment. Such detailed studies will provide sound ground to treat various infectious and harmful pathogen-based diseases cautiously. Biomolecules including peptides and proteins are well-known for their self-assembling capability to prepare nanostructures via non-covalent interactions (e.g., H-bonding, electrostatic, Π-Π stacking, and hydrophobic interactions) and are functioned as capping, stabilizing, and reducing agents for metal ions. Therefore, the biomolecule-based approach could be a good technique to prepare Ag nanoparticles that might be able to overcome the toxicity and stability highly associated with being used in healthcare systems. More intensive research is required to prepare Ag incorporating peptide and protein nanomaterials, with precise control over their formation, stability, biocompatibility, and other biophysical properties being needed for clinical studies.

## Figures and Tables

**Table 1:** The analysis of the search results is shown in Figure 1.

Documents Type	No. of Documents
Article	472
Review	44
Conference paper	19
Book chapter	9
Short survey	2
Conference review	1
Erratum	1
Note	1

## Data Availability

The data presented in this study are available in this study with copyright permissions.
